# The Effects of POWER Training in Young and Older Adults after Stroke

**DOI:** 10.1155/2016/7316250

**Published:** 2016-07-17

**Authors:** Jennifer L. Hunnicutt, Stacey E. Aaron, Aaron E. Embry, Brian Cence, Patrick Morgan, Mark G. Bowden, Chris M. Gregory

**Affiliations:** ^1^Department of Health Sciences and Research, College of Health Professions, Medical University of South Carolina, 77 President Street, MSC 700, Charleston, SC 29425, USA; ^2^Division of Physical Therapy, College of Health Professions, Medical University of South Carolina, 151-B Rutledge Avenue, MSC 962, Charleston, SC 29425, USA; ^3^Ralph H. Johnson VA Medical Center, 109 Bee Street, Charleston, SC 29401, USA

## Abstract

*Background*. Approximately 35,000 strokes occur annually in adults below the age of 40, and there is disappointingly little data describing their responses to rehabilitation. The purpose of this analysis was to determine the effects of Poststroke Optimization of Walking using Explosive Resistance (POWER) training in young (<40 years) and older (>60 years) adults and to describe relationships between training-induced improvements in muscular and locomotor function.* Methods*. Data was analyzed from 16 individuals with chronic stroke who participated in 24 sessions of POWER training. Outcomes included muscle power generation, self-selected walking speed (SSWS), 6-minute walk test, Fugl-Meyer motor assessment, Berg Balance Scale, and Dynamic Gait Index.* Results*. There were no significant differences between groups at baseline. Within-group comparisons revealed significant improvements in paretic and nonparetic knee extensor muscle power generation in both groups. Additionally, young participants significantly improved SSWS. Improvements in SSWS were more strongly associated with improvements in power generation on both sides in young versus older participants.* Conclusions*. Younger adults after stroke seem to preferentially benefit from POWER training, particularly when increasing gait speed is a rehabilitation goal. Future research should aim to further understand age-related differences in response to training to provide optimal treatments for all individuals following stroke.

## 1. Introduction

Although stroke is largely considered a condition of the aged, approximately 35,000 new strokes occur annually in individuals under the age of 40 years [1], an incidence that is three times that of new spinal cord injuries or diagnoses of multiple sclerosis. Despite this frequency, data describing the impact of rehabilitation on this growing cohort is absent, and a fundamental need exists to gain insight into the unique capabilities and benefits of rehabilitation for these young individuals. Training to improve lower extremity muscle power generation is an emerging intervention approach in both stroke and nonstroke rehabilitation trials. The potential impact of this training in individuals after stroke is reflected by the facts that significant losses in muscular power generation occur as a direct result of the stroke as well as from the natural aging process, with power declining more rapidly than muscle strength [2, 3]. These declines in muscle power generation negatively impact mobility, limiting propulsion of the body forward during walking and contributing to observed slow walking speeds. Given that slow walking is one of the primary complaints following stroke [4], it is necessary to implement interventions that effectively restore muscular function and translate to improvement in locomotor performance.

A training program entitled Poststroke Optimization of Walking using Explosive Resistance (POWER) has been developed to target muscle power generation dysfunction in individuals aged 18 to 75 years. This training program has been previously documented as a feasible intervention to improve muscular and locomotor function in this group [5]. However, the differential effects of this intervention in young versus older adults have not been explored. The purpose of this secondary analysis is to compare the effects of POWER training on changes in muscle power generation and locomotor function in young (<40 years) and older (>60 years) adults following stroke as well as to describe relationships between training-induced improvements in muscular and locomotor function.

## 2. Methods

### 2.1. Participants

A convenience sample of sixteen individuals (young = 6; older = 10) with chronic stroke (≥6 months) who completed 24 sessions of POWER training were included in this secondary analysis of a larger, ongoing trial investing the effects of POWER training on muscular and locomotor function. Inclusion criteria were the following: (1) ages 18–40 or 60–75 years, (2) residual paresis in lower extremity (Fugl-Meyer lower extremity motor score <34), (3) ability to walk 10 meters without support from another individual, and (4) lower than normal self-selected walking speed (SSWS) (<1.2 m/s). Individuals were excluded if they had history of preexisting neurological, psychiatric, or orthopedic problems that would hinder ability to complete aspects of testing or training. All participants that met inclusion criteria were required to complete an exercise tolerance test and be cleared for participation by the study cardiologist. This study was approved by the Institutional Review Board of the Medical University of South Carolina, and participants provided written informed consent prior to participation.

### 2.2. Intervention

Subjects completed 24 sessions of POWER training, as previously described [5]. Exercises included leg press, calf raises, and jump training all performed on a supine exercise device (Shuttle Pro MVP; Shuttle Systems Inc., Glacier, WA). Subjects were asked to perform the concentric phase of each exercise as quickly as possible and exercise intensity (i.e., resistance and number of repetitions) was progressed throughout the duration of the intervention and as tolerated by each individual. The number of sets of each exercise performed during a training session ranged from two to three, and the number of repetitions ranged from eight to twenty, depending on the goals for progression in the given session. Unilateral training was performed, the goal being to maximize the gains possible in each leg as opposed to training the paretic leg to reduce interlimb discrepancies. Task-specific training included sit-to-stands and step-ups and was progressed by lowering chair height, increasing step height, and adding a weighted vest, as tolerated by the individual. Subjects also completed repeated 10-meter trials of fast walking training (10 trials per session) at a minimum of 125% of SSWS to emphasize power generation within the task.

### 2.3. Outcome Measures

#### 2.3.1. Overground Walking

Participants walked at their SSWS measured using a GAITRite Portable Walking System (GAITRite*™*, CIR Systems, Inc., Sparta, New Jersey). Three trials were recorded and averaged for analyses prior to and after the training intervention. Participants who used orthotic devices were provided Aircasts® to wear during testing and were permitted to use assistive devices as needed.

#### 2.3.2. Muscle Power

Peak muscle power generation of the knee extensors was assessed during isotonic contractions on the Biodex isokinetic dynamometer (Biodex Medical Systems Inc., Shirley, New York), using an external resistance set at approximately 40% of their maximum voluntary isometric contraction. Differences in lower extremity maximal contractile velocity have been reported to occur against this external load (e.g., 40% 1 RM) and are most closely associated with gait velocity in older individuals [6]. Each test was repeated five times, and the peak velocity was used to calculate muscle power generation. All participants were instructed to generate torque as fast as possible during testing.

#### 2.3.3. Clinical Assessments

Clinical assessments included Fugl-Meyer lower extremity motor score (FMA-LE) [7], Berg Balance Scale (BBS) [8], Dynamic Gait Index (DGI) [9], and 6-minute walk test (6MWT) [10]. All assessments were performed by a licensed physical therapist prior to and following the intervention.

### 2.4. Data Analysis

Within-group changes were assessed using paired sample *t*-tests, while between-group comparisons were made using independent sample *t*-tests. Associations between training-induced improvements in muscular and locomotor function were determined using Pearson product moment correlation coefficients. For all analyses, level of significance was set at *α* < 0.05. Adjustments to level of significance were made for multiple comparisons using Bonferroni correction.

## 3. Results

At baseline there were no significant differences in clinical assessments, SSWS, or muscle power generation between young and older groups (Table 1). For the entire sample, baseline SSWS moderately correlated with baseline muscle power generation on the paretic side (*r* = 0.42). Following training, both young and older participants significantly improved paretic and nonparetic knee extensor muscle power generation.

**Figure 1:**
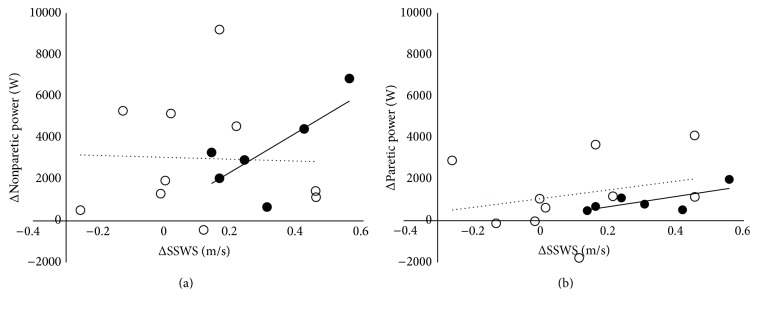
Associations between change (Δ) in SSWS (self-selected walking speed) and muscle power generation on nonparetic (a) and paretic (b) sides. Closed circles (solid line) are young subjects; open circles (dotted line) are older subjects.

Young subjects significantly improved SSWS (*p* = 0.003), while improvements in SSWS in older subjects did not reach statistical significance (*p* = 0.09). No between-group (young versus older) differences in response to training occurred. Interestingly, changes in knee extensor muscle power generation appear more strongly associated with improvements in SSWS on both the paretic (*r* = 0.71 versus 0.27) and nonparetic (*r* = 0.72 versus −0.04) sides in young subjects compared to older subjects (Figure 1).

## 4. Discussion

These results demonstrate that 24 sessions of POWER training can significantly improve lower extremity muscle power generation in both young and older individuals following stroke. Training significantly improved locomotor function in young, but not older, poststroke subjects, even though both groups walked at similar gait speeds prior to training. Despite the relatively short duration of POWER training, young participants reached clinically meaningful improvements in SSWS (0.31 m/s) [11, 12] and 6MWT (64 m) [10], while older subjects did not (0.1 m/s and 2 m, resp.).

Individuals with poststroke hemiparesis experience deficits in the force generating capacity of muscles, in combination with substantial muscle weakness, that affect complex movement patterns such as walking. Individuals with stroke can be compared to their neurologically healthy aged counterparts, who also experience pronounced velocity dependent muscular deficits that limit mobility [6] and increase fall risk [13]. More specifically, lower extremity muscle power generation is more strongly correlated with mobility in functional tasks compared to muscular strength [14]. Following stroke, deficits in strength and power of the paretic lower extremity explain ~67% of the variance in walking speeds [15]. Our data provide similar support for a relationship between paretic muscle function and gait speed with a moderate correlation (*r* = 0.42) for the entire sample at baseline. This is only slightly lower than correlations that have been previously reported in the stroke literature [16, 17]. Importantly, support for a more mechanistic relationship between lower extremity power generation and gait speed is suggested in our young subjects, as evidenced by the association between improvements in muscular and locomotor function in this group. Strong associations between improvements in muscle power generation and walking speeds have also been demonstrated in the subacute phase of stroke [17]. Interestingly, our young participants have a greater mean time since stroke (59.7 months) compared to the older group (27.4 months); thus we can conclude that chronicity is not driving the lack of significant gait speed improvements in the older group.

The results of the present study are consistent with studies in neurologically healthy older adults, in which POWER training enhanced functional outcomes when compared to traditional strength training [18]. It is yet to be determined if POWER training may be a more appropriate intervention than traditional strength training following stroke, as these two interventions have not been compared in a randomized controlled trial. With respect to the effects of age on rehabilitation, data from the LEAPS trial [19] show that younger age is a significant predictor of response to locomotor training. Although older subjects did significantly improve muscle power generation, our data show preferential locomotor responses to POWER training in young subjects. With slow walking being one of the primary complaints following stroke [4], the potential to restore muscle power generation in these individuals provides an appropriate rehabilitation goal when targeting improvements in locomotion. Future research should be directed at gaining more in-depth understanding of age-related differences in response to POWER training, specifically how POWER training can be designed to optimize functional gains in individuals across age groups.

A few limitations are worthy of mentioning in this analysis. Sample size, particularly of the young group, limits generalizability of this study. Our sample size also prohibits testing of normality and thus our analyses are limited in their scope. This is not uncommon in studies using convenience samples and the data should be interpreted with this in mind. Additionally, the cohort in this analysis consisted of high-functioning stroke participants, as seen by high pretraining scores on clinical assessments, which also limits generalizability to individuals of varying functional statuses. This may also explain the lack of significant improvements in clinical assessments (FMA-LE, BBS, and DGI) from pre- to posttraining caused by the existing ceiling effect within the data. Lastly, the inclusion of both resistance training exercises and task-specific exercises impedes our ability to attribute improvements in locomotor performance to one or the other. However, the study intervention was designed with a clinical application in mind and implicates that both resistance training and task-specific training should be utilized.

In conclusion, it is essential to provide evidence-based therapies that address the particular needs of both young and older individuals following stroke, offering unique challenges to clinicians treating this population. The results of this study particularly support the implementation of power training for young individuals with chronic stroke, especially if increasing gait speed is a rehabilitation goal.

## Figures and Tables

**Table 1:** Participant demographics and outcome measures (mean ± standard deviation).

	Young (*n* = 6)		Old (*n* = 10)	
	Pre	Post	Pre	Post
*Demographics*				
Age (years)	28.2 ± 5.2	—	65.8 ± 4.2	—
Time since stroke (mos)	59.7 ± 46.7	—	27.4 ± 29.0	—
FMA-LE (0–34)	18.8 ± 4.0	20.7 ± 5.8	22.3 ± 7.1	23.1 ± 8.4
BBS (0–56)	44.0 ± 10.2	46.5 ± 10.1	43.7 ± 10.9	42.6 ± 11.1
DGI (0–24)	15.2 ± 4.9	16.3 ± 4.0	15.6 ± 5.2	14.3 ± 6.1

*Outcome measures*				
SSWS (m/s)	0.55 ± 0.32	0.86 ± 0.42^*∗*^	0.67 ± 0.42	0.77 ± 0.45
6MWT (m)	310 ± 157	374 ± 123	265 ± 143	267 ± 152
PKP (W/kg)	77.7 ± 64.9	88.9 ± 65.3^*∗*^	57.4 ± 31.1	72.3 ± 38.3^*∗*^
NPKP (W/kg)	174.9 ± 63.5	221.3 ± 78.4^*∗*^	131.5 ± 29.4	200.7 ± 58.0^*∗*^

^*∗*^Significant within-group difference compared with pretraining.

FMA-LE: Fugl-Meyer assessment-lower extremity motor score, BBS: Berg Balance Scale, DGI: Dynamic Gait Index, SSWS: self-selected walking speed, 6MWT: 6-minute walk test, PKP: paretic knee power, and NPKP: nonparetic knee power.
